# A Clozapine-Responsive GPCR-Based Gene Switch for Pharmacological Control of Gene Expression in Mammalian Cells and In Vivo

**DOI:** 10.3390/ijms27083381

**Published:** 2026-04-09

**Authors:** Guanyang Chen, Shiting Li, Peng Bai

**Affiliations:** 1 Department of Laboratory Medicine, the First Affiliated Hospital, Sun Yat-sen University, Guangzhou 510080, China; 2 Institute of Precision Medicine, the First Affiliated Hospital, Sun Yat-sen University, Guangzhou 510080, China

**Keywords:** synthetic gene switches, synthetic biology, gene and cell therapy

## Abstract

The safe and precise regulation of therapeutic gene expression remains a major challenge for mammalian synthetic biology and cell-based therapies. Many existing inducible systems rely on non-mammalian regulatory components or ligands with limited clinical compatibility. Designer receptors exclusively activated by designer drugs (DREADDs) offer a human G protein-coupled receptor (GPCR)-based framework for pharmacological control of intracellular signaling, yet their application as clinically relevant gene-regulation platforms remains underexplored. Here, we report a clozapine-responsive gene switch that couples a designer GPCR to signaling-dependent transcriptional control. By linking clozapine-activated receptors to cyclic adenosine monophosphate (cAMP)- or calcium-responsive synthetic promoters, receptor activation is converted into robust transgene expression across a broad dynamic range, with sensitivity to sub-nanomolar to low-nanomolar clozapine concentrations. In vivo, alginate-encapsulated reporter cells implanted in C57BL/6J mice responded to systemic or local clozapine administration with efficient secretion of a reporter protein, achieving robust induction at low daily doses (0.3 mg/kg) following either oral administration or local delivery. Together, these results establish a human GPCR-based clozapine-responsive gene switch that integrates regulation by a clinically used small molecule with modular transcriptional outputs, providing an additional approach for pharmacologically controllable gene expression in mammalian cells and in vivo.

## 1. Introduction

Precise and externally controllable regulation of transgene expression is a fundamental requirement for the safe implementation of gene- and cell-based therapies [1,2]. Synthetic biology has enabled the development of diverse inducible gene-control systems that allow therapeutic outputs to be activated, modulated, or terminated on demand [1,3,4]. However, despite substantial advances in circuit design and regulatory architectures, the translational applicability of many existing systems remains limited [5,6]. A central challenge lies not only in achieving robust inducibility but also in identifying regulatory inputs that are compatible with human physiology and clinical use [5,6].

From a translational perspective, regulatory inputs ideally should be human-derived and responsive to small molecules with favorable pharmacokinetic and safety profiles [7,8,9]. Accordingly, engineering human-derived regulatory components has attracted increasing interest as a strategy to reduce immunogenicity and improve physiological compatibility, although achieving stringent external controllability remains challenging [9]. A key limitation of fully human regulatory elements is their intrinsic sensitivity to endogenous ligands, such as hormones, neurotransmitters, or metabolites, which can lead to unintended basal activation and compromise external control [6,8]. This trade-off between human origin and orthogonality presents a major challenge to the development of clinically viable gene-control systems [6,8].

Designer receptors exclusively activated by designer drugs (DREADDs) provide a unique solution to this challenge by combining a fully human G protein-coupled receptor (GPCR) scaffold with engineered ligand selectivity achieved through targeted mutagenesis [10,11]. By reducing responsiveness to endogenous neurotransmitters while introducing high affinity for otherwise pharmacologically inert ligands, DREADDs largely decouple human receptor architecture from endogenous signaling backgrounds [10,11]. This design enables precise, externally controlled activation of defined intracellular signaling pathways while reducing responsiveness to endogenous ligands, thereby overcoming a key limitation of conventional human-derived regulatory elements [10,11].

Among the available DREADD ligands, clozapine has emerged as an effective pharmacological input due to its high affinity for multiple DREADD variants at nanomolar concentrations [10]. Although clozapine is a clinically approved antipsychotic with well-characterized dose-dependent effects at therapeutic levels, accumulating evidence indicates that ultra-low doses are sufficient to activate designer receptors without eliciting overt behavioral or systemic consequences [10]. These properties position clozapine as a clinically used and systemically deliverable small-molecule trigger for engineered receptor systems.

To date, DREADDs have been used predominantly as tools for modulating cellular excitability and signaling in neuroscience and physiology [11,12,13]. Beyond neuroscience applications, chemogenetic approaches based on DREADDs have been proposed as broadly applicable tools for therapeutic and translational control of cellular function [14]. In contrast, their potential as modular inputs for programmable control of gene expression has received comparatively limited attention. Whether DREADD-mediated signaling can be systematically harnessed to drive precise and tunable transcriptional programs remains an open question, particularly in the context of gene- and cell-based therapeutic applications. Previous studies have demonstrated that GPCR signaling can be rewired to control transcriptional outputs in mammalian cells, highlighting the potential of receptor-based gene circuits in whole-cell systems [15].

Tet-based systems have been widely used for robust transcriptional regulation, but their dependence on heterologous regulatory components remains an important consideration for translational applications [16,17]. Optogenetic approaches enable high spatiotemporal precision but require dedicated hardware for activation. Other small-molecule-responsive mammalian gene circuits have further illustrated the potential of pharmacological control in whole-cell systems [7,8]. GPCR-based chemogenetic systems provide a way to connect extracellular drug inputs with intracellular signaling and downstream transcriptional regulation, offering a relevant framework for the development of human-derived and pharmacologically controlled gene switches.

In this study, we sought to extend the utility of DREADD-based receptor systems beyond modulation of cellular activity toward programmable regulation of gene expression. To achieve this, we designed a receptor–promoter gene-control architecture that directly couples clozapine-activated GPCR signaling to pathway-dependent transcriptional outputs. Rather than introducing exogenous transcription factors, receptor activation is transduced through endogenous second-messenger pathways and decoded by synthetic promoters responsive to defined signaling states, enabling tight integration between pharmacological input and transcriptional control.

This modular design allows a human-derived receptor scaffold and clinically used pharmacological input to be flexibly interfaced with distinct transcriptional programs by selecting appropriate signaling-responsive promoter elements. Such an approach preserves key pharmacological features of DREADDs, including engineered ligand selectivity, nanomolar sensitivity, and compatibility with systemic pharmacological delivery, while enabling controllable and tunable regulation of transgene expression. By validating this strategy in mammalian cells and in vivo using implanted reporter cells, our work suggests a framework for leveraging clinically compatible GPCR signaling as a regulatory input for gene-control systems.

## 2. Results

### 2.1. Design and Selection of Receptor–Promoter Combinations for Clozapine-Responsive Gene Regulation

**Figure 1:**
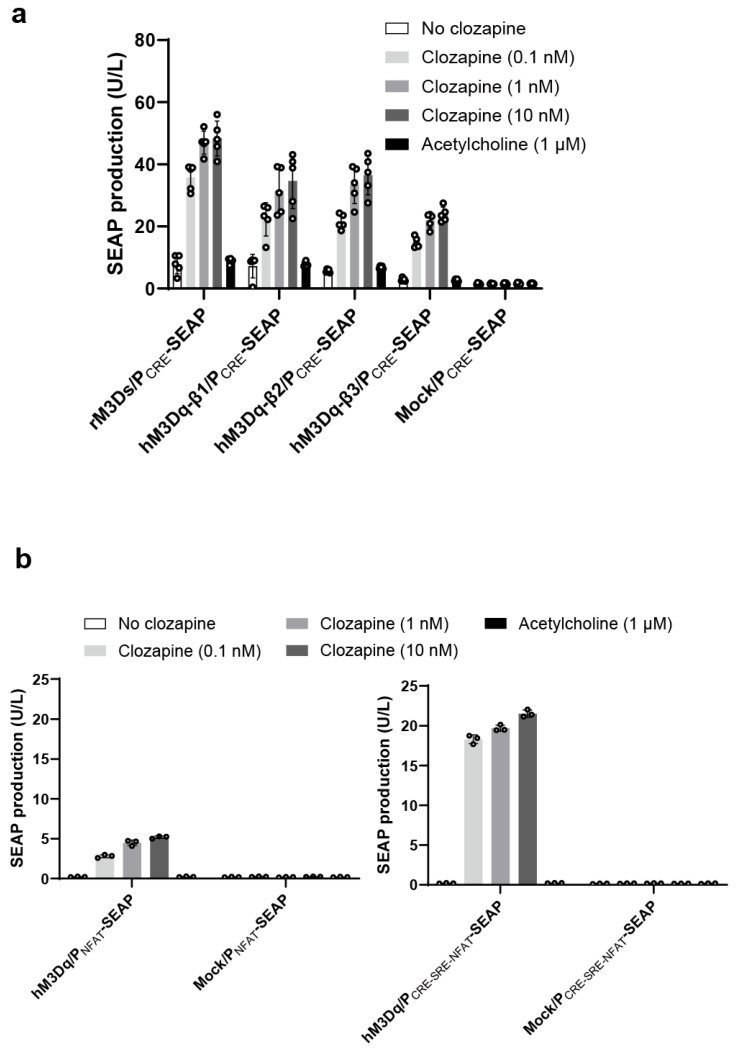
Screening of designer receptors exclusively activated by designer drugs (DREADD) receptor–promoter configurations for clozapine-responsive transcription. (**a**) Cyclic adenosine monophosphate (cAMP)-responsive transcription mediated by DREADD-based and chimeric receptors. HEK293T cells were transiently co-transfected with a cAMP-responsive reporter (P_CRE_-SEAP) and the indicated DREADD receptor constructs, including the prototypical Gs-coupled DREADD receptor rM3Ds and chimeric receptors in which the extracellular ligand-binding domain of hM3Dq was fused to the intracellular domains of human β1-, β2-, or β3-adrenergic receptors. Cells co-transfected with the reporter plasmid and a green fluorescent protein (GFP) expression vector served as controls. Cells were stimulated with clozapine or the endogenous muscarinic agonist acetylcholine at the indicated concentrations for 24 h, and secreted alkaline phosphatase (SEAP) activity in the culture supernatant was quantified. Data are presented as mean ± standard deviation (s.d.); n = 3–5 biologically independent samples. (**b**) Calcium-responsive transcription driven by hM3Dq-based receptor–promoter configurations. HEK293T cells expressing hM3Dq were co-transfected with SEAP reporters driven by either a nuclear factor of activated T cells (NFAT)-responsive promoter (P_NFAT_-SEAP) or a composite calcium-responsive promoter integrating cAMP response element (CRE), serum response element (SRE), and NFAT response elements (P_CRE-SRE-NFAT_-SEAP). Cells co-transfected with the reporter plasmid and a GFP expression vector served as controls. Cells were stimulated with clozapine or acetylcholine for 24 h under identical conditions, and SEAP secretion was measured to quantify calcium-dependent transcriptional responses. Data are presented as mean ± s.d.; n = 3 biologically independent samples.

The prototypical Gs-coupled DREADD receptor rM3Ds is a well-established tool for activating the cyclic adenosine monophosphate (cAMP) signaling pathway; however, it is derived from a rat muscarinic receptor scaffold, which limits its suitability for human-oriented gene-control applications. In contrast, hM3Dq is derived from the human muscarinic M3 receptor and has been widely used to activate G protein αq (Gαq)-mediated calcium signaling. To establish a clozapine-responsive gene-regulation system based on human receptor components while enabling access to distinct intracellular signaling pathways, we evaluated a panel of DREADD-based receptors, including chimeric designs, that couple clozapine-dependent receptor activation to transcriptional outputs. Specifically, we examined chimeric receptors in which the extracellular ligand-binding domain of hM3Dq was fused to the intracellular domains of human β1 [13] or β2 [12] adrenergic receptors, designs that have been previously reported to redirect signaling toward the cAMP pathway in response to designer ligands. In addition, we constructed a new chimeric receptor by fusing the hM3Dq extracellular domain to the intracellular domain of the human β3-adrenergic receptor. Whereas hM3Dq–β1 and hM3Dq–β2 chimeric receptors have been previously characterized using clozapine-N-oxide (CNO) as the activating ligand, the clozapine responsiveness of these receptor designs, particularly in the context of transcriptional readout, has not been systematically evaluated. We therefore tested all receptor variants for clozapine-induced activation in HEK293T cells using a cAMP-responsive reporter construct (P_CRE_-SEAP), in which secreted alkaline phosphatase (SEAP) expression was driven by a promoter containing cAMP response elements (P_CRE_) [18] (Figure 1a).

In parallel, we assessed the ability of hM3Dq-based receptors to drive calcium-dependent transcription by pairing them with synthetic calcium-responsive promoters. Two promoter architectures were compared: a nuclear factor of activated T cells (NFAT)-responsive reporter containing NFAT elements alone (P_NFAT_-SEAP) and a composite promoter integrating cAMP response element (CRE), serum response element (SRE), and NFAT response elements (P_CRE-SRE-NFAT_-SEAP) [19,20], designed to capture convergent calcium-dependent transcriptional signals [20] (Figure 1b). Comparative screening of different receptor–promoter combinations at fixed low nanomolar clozapine concentrations (0.1, 1, and 10 nM) yielded distinct activation amplitudes. While cAMP-responsive promoter configurations driven by hM3Dq–β-adrenergic chimeric receptors exhibited measurable induction, pairing hM3Dq with the composite calcium-responsive promoter (P_CRE-SRE-NFAT_-SEAP) yielded a higher fold induction under the tested conditions (Figure 1a,b and Figure S2). Based on these screening results, this receptor–promoter configuration was selected for subsequent characterization.

To examine whether HEK293T cells express endogenous NFAT family members, we analyzed *NFATC1–C4* expression by quantitative PCR (qPCR). All four transcripts were detectable in HEK293T cells (Figure S1). We next examined whether the transcriptional response of the selected P_CRE-SRE-NFAT_ reporter involved canonical NFAT signaling by treating cells with the calcineurin inhibitor cyclosporin A (CsA). CsA markedly suppressed clozapine-induced reporter activation, whereas output from the constitutive simian virus 40 (SV40) promoter-driven SEAP reporter (P_SV40_-SEAP) showed little apparent change under the tested conditions (Figure S3).

Additional specificity analyses, including an acetylcholine dose–response experiment and testing of representative neurotransmitters, showed no appreciable activation under the examined conditions (Figures S4 and S5). For reference, the selected hM3Dq/P_CRE-SRE-NFAT_ configuration was compared with a Tet-On system and a constitutive P_SV40_-SEAP control under the same experimental conditions (Figure S6). Under the tested conditions, the selected configuration showed relatively low basal activity. The switch was also validated in additional mammalian cell contexts, including CHO-K1, COS-1, and hMSC-TERT-derived cells (Figure S7).

### 2.2. Dose-Dependent Activation of the Selected Gene Switch by Clozapine

To quantitatively characterize the concentration dependence of the selected gene switch, HEK293T cells were transiently co-transfected with the hM3Dq expression plasmid and the calcium-responsive SEAP reporter (P_CRE-SRE-NFAT_-SEAP) and exposed to a broad range of clozapine concentrations (0.01–100 nM). Reporter activity was measured after ligand stimulation using secreted alkaline phosphatase (SEAP) as a readout of transcriptional activation.

**Figure 2:**
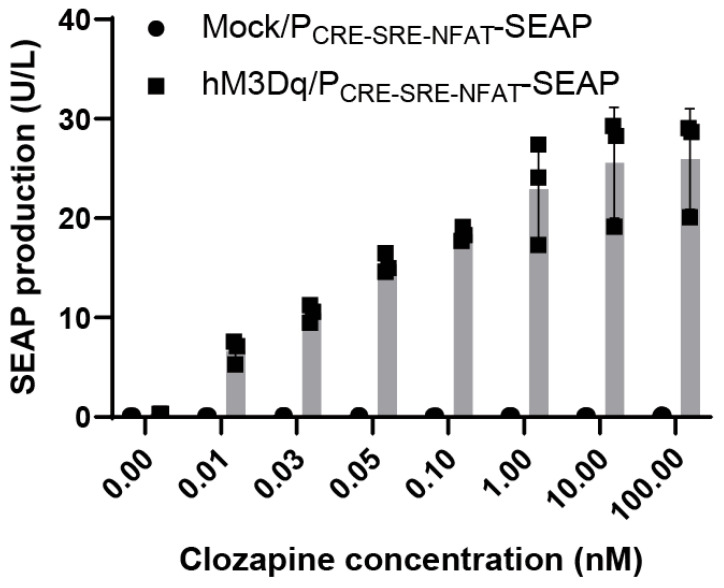
Dose–response characterization of clozapine-induced gene expression. HEK293T cells were transiently co-transfected with the hM3Dq expression plasmid and a calcium-responsive SEAP reporter (P_CRE-SRE-NFAT_-SEAP), followed by stimulation with increasing concentrations of clozapine (0.01–100 nM) for 24 h. Secreted alkaline phosphatase (SEAP) activity in the culture supernatant was quantified to measure ligand-dependent transcriptional output. Cells co-transfected with the reporter plasmid and a GFP expression vector were included as controls and assayed in parallel. Data are presented as mean ± s.d.; n = 3 biologically independent samples.

Clozapine induced gene expression in a concentration-dependent manner across the tested range (Figure 2). Detectable activation was observed at 0.01 nM, with robust induction at 0.1 nM. Reporter output increased steeply between 0.01 and 1 nM and reached near-maximal levels at approximately 1 nM clozapine. Further increases in ligand concentration up to 100 nM did not result in substantial additional activation, consistent with an apparent plateau in activation within the tested range. Cells co-transfected with the reporter plasmid and a green fluorescent protein (GFP) expression vector did not exhibit clozapine-dependent activation, consistent with transcriptional induction being dependent on the presence of the hM3Dq receptor (Figure 2).

Notably, the effective concentrations required for gene-switch activation fall within the sub-nanomolar to low-nanomolar range and are substantially lower than plasma concentrations reported during clinical clozapine treatment, suggesting that only minimal systemic exposure may be sufficient for switch activation.

### 2.3. Temporal Characteristics of Clozapine-Induced Gene Expression

To examine the temporal dynamics of clozapine-induced gene expression, HEK293T cells transiently co-transfected with the hM3Dq expression plasmid and the P_CRE-SRE-NFAT_-SEAP reporter were exposed to clozapine for defined durations. SEAP secretion was subsequently measured to assess the kinetics of transcriptional activation.

**Figure 3:**
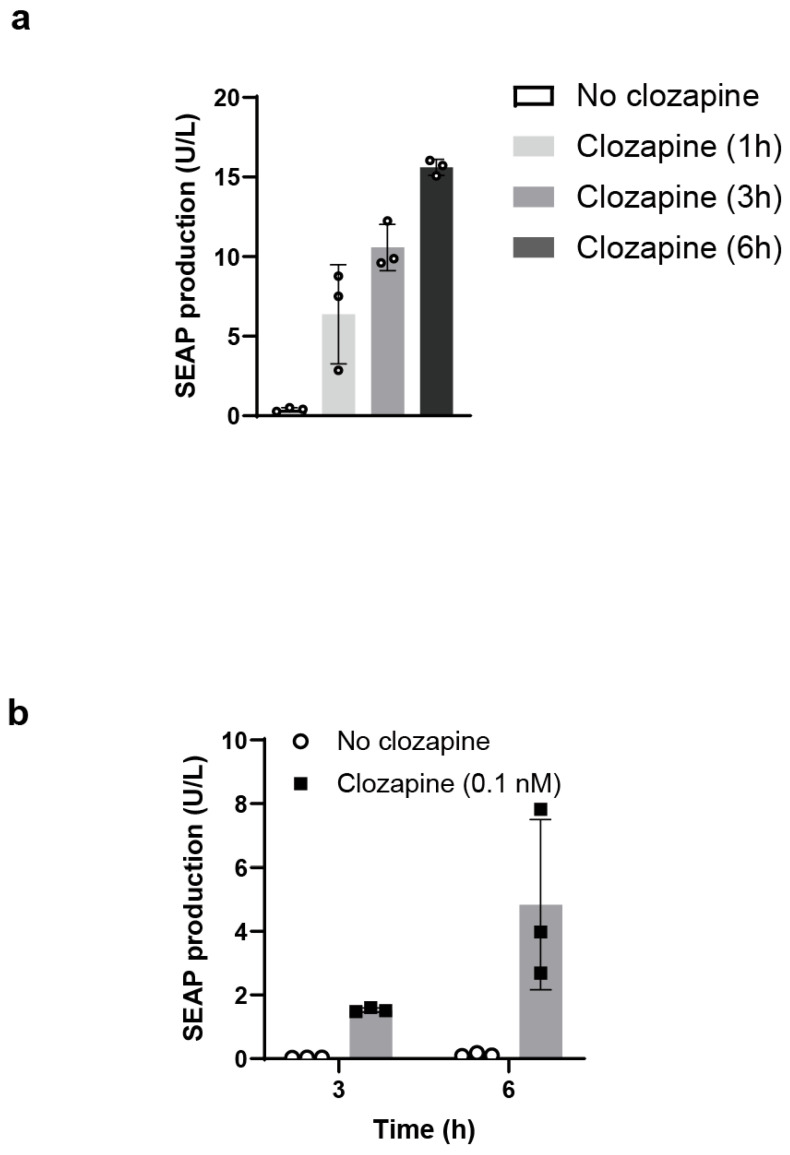
Temporal characteristics of clozapine-induced transcriptional activation. (**a**) Duration-dependent effects of clozapine exposure on transcriptional output. HEK293T cells were transiently co-transfected with hM3Dq and a calcium-responsive SEAP reporter (P_CRE-SRE-NFAT_-SEAP), then stimulated with clozapine for the indicated durations (0, 1, 3, or 6 h). After stimulation, the culture medium was replaced with ligand-free medium, and SEAP activity in the supernatant was quantified at a fixed total culture time of 24 h. Data are presented as mean ± s.d.; n = 3 biologically independent samples. (**b**) Time course of SEAP secretion following clozapine stimulation. HEK293T cells transiently co-transfected with hM3Dq and P_CRE-SRE-NFAT_-SEAP were stimulated with 0.1 nM clozapine (or left unstimulated), and SEAP activity in the culture supernatant was measured at the indicated time points (3 and 6 h). Data are presented as mean ± s.d.; n = 3 biologically independent samples.

Short-term clozapine stimulation was able to initiate transgene expression. Exposure for 1 h resulted in detectable SEAP secretion (Figure 3a). Substantial reporter output was observed within 3 h after stimulation, consistent with rapid coupling between receptor activation and transcriptional response (Figure 3b).

### 2.4. In Vivo Activation of the Clozapine-Responsive Gene Switch Using Encapsulated Cells

To evaluate whether the clozapine-responsive gene switch functions in vivo, HEK293T cells transiently co-transfected with the hM3Dq expression plasmid and the P_CRE-SRE-NFAT_-SEAP reporter were encapsulated in alginate-based microcapsules and implanted into mice. This encapsulation strategy physically confines engineered cells while allowing diffusion of small-molecule ligands and secreted reporter proteins.

**Figure 4:**
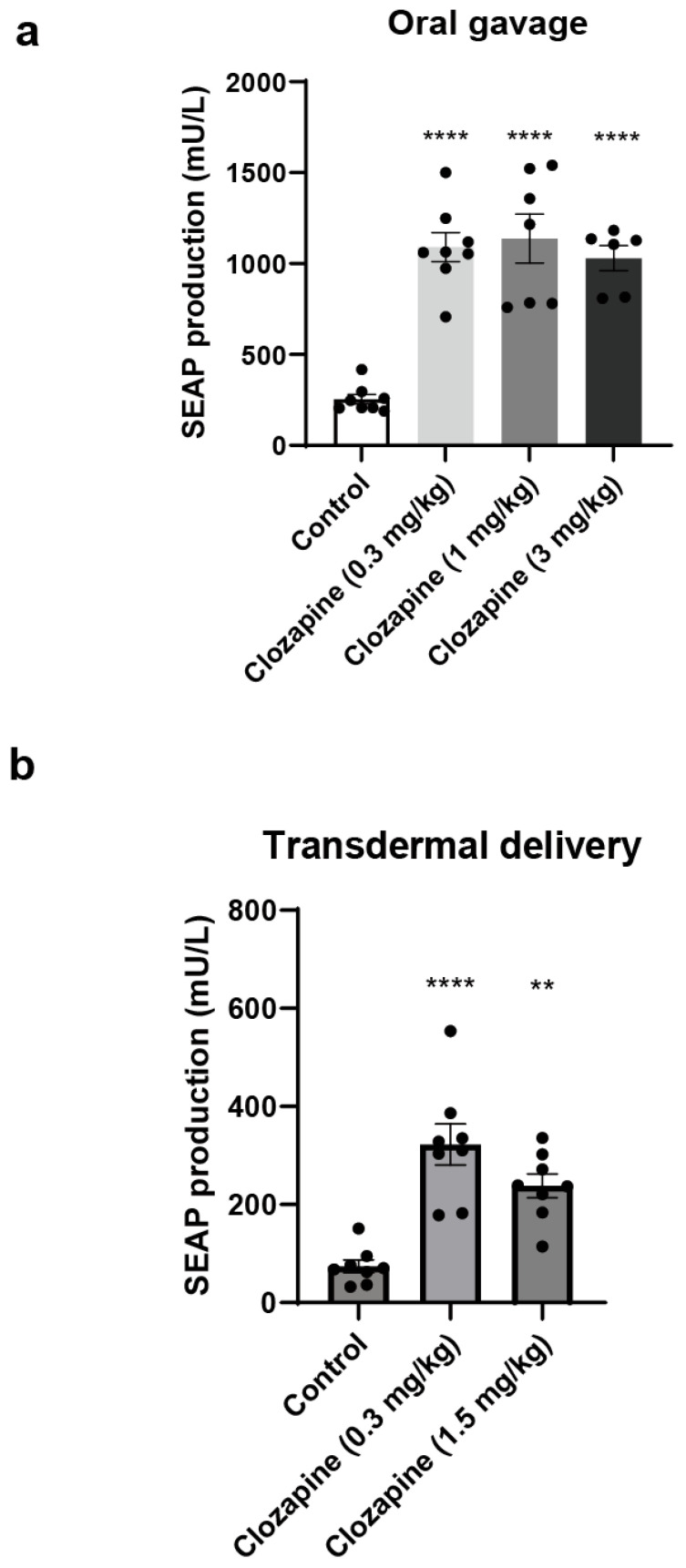
In vivo activation of the clozapine-responsive gene switch by systemic and local drug administration. Alginate-encapsulated HEK293T reporter cells were generated by transient co-transfection with hM3Dq and a calcium-responsive SEAP reporter (P_CRE-SRE-NFAT_-SEAP). For systemic administration, encapsulated cells were delivered by intraperitoneal (i.p.) injection, and clozapine was administered by oral gavage (0.3, 1, or 3 mg/kg/day) once daily for two consecutive days (**a**). For local administration, encapsulated cells were implanted subcutaneously (s.c.), and clozapine was applied topically at the implantation site at doses of 0.3 or 1.5 mg/kg once daily for two consecutive days (**b**). Blood samples were collected 48 h after cell implantation, and serum SEAP levels were quantified using a chemiluminescent SEAP assay (reported as mU/L). Each dot represents one mouse; bars indicate mean ± standard error of the mean (s.e.m.). Statistical significance was determined by one-way analysis of variance (ANOVA) with Dunnett’s multiple comparisons test versus control (** *p* < 0.01, **** *p* < 0.0001). n = 6–8 mice per group.

Following intraperitoneal implantation of encapsulated cells, clozapine was administered systemically by oral gavage at the indicated doses (0.3, 1, or 3 mg/kg) once daily for two consecutive days. Serum SEAP levels were measured 48 h after implantation to assess in vivo activation of the implanted gene switch. Clozapine treatment resulted in increased circulating SEAP levels compared with vehicle-treated control groups (Figure 4a). Reporter induction was observed at low daily doses, including 0.3 mg/kg/day, which are substantially lower than doses commonly used for established therapeutic indications. Within the tested dosing range (0.3–3 mg/kg/day), SEAP induction levels were comparable across doses, indicating that gene-switch activation could be achieved at low clozapine doses.

To assess whether local drug delivery could also activate the implanted gene switch, encapsulated cells were implanted subcutaneously, and clozapine was applied topically at the implantation site once daily for two consecutive days. Local administration resulted in a significant increase in reporter secretion (Figure 4b), indicating that clozapine applied at the implantation site was sufficient to reach and activate the encapsulated cells.

Together, these results demonstrate that the clozapine-responsive gene switch enables effective pharmacological control of transgene expression from implanted, encapsulated cells in vivo, with robust activation achievable at low clozapine doses.

## 3. Discussion

In this study, we establish a clozapine-responsive gene-control system that repurposes a human-derived designer GPCR as a pharmacological input for modular regulation of transgene expression. By coupling hM3Dq-mediated signaling to synthetic, pathway-responsive promoters, the system converts ligand engagement into tunable transcriptional outputs without the need for non-mammalian transcription factors or virus-derived regulatory proteins. Rather than introducing a new gene-switching mechanism, this work deliberately prioritizes pharmacological compatibility and external controllability as primary design constraints, addressing a central challenge in the development of clinically compatible gene-regulation platforms.

A defining feature of the system is its high sensitivity to clozapine, enabling robust gene-switch activation at sub-nanomolar concentrations in vitro and at low systemic doses in vivo. Notably, effective activation was achieved at mouse doses that correspond to the lower end of clinically relevant exposure ranges, rather than therapeutic maintenance levels. From a practical perspective, the observation that robust activation can be achieved at low doses may help reduce ligand burdens and simplify the pharmacological application of the system.

Beyond sensitivity, the clozapine-responsive gene switch exhibits rapid activation kinetics, allowing transcriptional programs to be initiated through transient ligand exposure. Together with its functionality in encapsulated cells in vivo and compatibility with both systemic and local drug delivery, these properties position the system as a practical framework for pharmacologically controllable gene expression. Encapsulation of engineered cells has been widely explored as a strategy to implement controllable whole-cell systems in vivo, enabling physical containment, retrievability, and external regulation of cellular function [21]. While demonstrated here using a reporter payload, the underlying receptor–promoter architecture is modular and can, in principle, be extended to diverse therapeutic genes and cell-based applications.

The clozapine-responsive system described here emphasizes the use of human-derived receptor scaffolds combined with clinically established small-molecule control. Whereas prior platforms frequently rely on heterologous regulatory components [16,17,22], synthetic ligands without clinical precedence [23], or complex multi-component assemblies [3], the present design employs a human-derived receptor scaffold paired with a clinically used ligand that has well-characterized pharmacology and systemic bioavailability. This distinction does not imply superior transcriptional performance, but rather highlights a complementary design space in which pharmacological familiarity and translational plausibility are prioritized alongside modularity and controllability. Within the broader landscape of inducible gene-control systems, this design may be particularly relevant in settings where pharmacological administration is more practical than hardware-dependent actuation, while reduced responsiveness to endogenous inputs remains an important design consideration.

Moreover, while DREADDs have been extensively applied for modulating cellular activity, particularly in neuroscience, their systematic use as inputs for programmable gene regulation has remained comparatively limited. DREADDs provide a compelling paradigm in which targeted mutagenesis is used to reduce responsiveness to endogenous ligands while conferring selective sensitivity to an exogenously administered small molecule. By integrating DREADD signaling directly with synthetic transcriptional decoding modules, the present work extends the functional scope of these receptors from acute modulation of cell signaling toward sustained and externally controllable gene expression. Accordingly, the clozapine-responsive gene switch is particularly suited for applications requiring short-term, tightly controlled activation of gene expression, such as implanted or encapsulated cell therapies under clinical supervision. The system therefore provides an additional, pharmacologically oriented option within the broader toolkit of mammalian synthetic biology.

At the same time, clozapine is not an ideal control ligand for all applications. Its known off-target pharmacology and the potential constraints associated with repeated or long-term administration may limit its broader translational applicability [24]. However, the low concentrations and doses sufficient for switch activation in the present study may help mitigate ligand burden. Accordingly, the hM3Dq–clozapine pair is best regarded as a practical and clinically informed starting point, whereas the underlying receptor–promoter framework remains amenable to future substitution with alternative pharmacological inputs.

Several limitations of the present study merit consideration. First, the gene-control system was primarily characterized in HEK293T cells using a reporter payload. HEK293T cells are widely used as a standardized benchmark for validating gene-switch architectures and pharmacological control strategies, providing a robust and reproducible platform for initial system-level evaluation; however, this setting does not capture cell-type–specific contexts relevant to therapeutic applications. Although supplementary experiments in additional mammalian cell contexts support transferability beyond HEK293T, broader validation in clinically relevant cell types and with functional therapeutic transgenes will still be necessary. Future studies will therefore be required to assess the performance of the clozapine-responsive gene switch in clinically relevant cell types and with functional therapeutic transgenes.

Second, although the specificity analyses showed no appreciable activation by acetylcholine or the additional endogenous neurotransmitters tested under the examined conditions, the present study does not rule out all possible endogenous or context-dependent inputs. The current data therefore support functional insulation from the tested ligands, rather than absolute orthogonality in all biological settings. Likewise, although the added pharmacological and reporter-component analyses strengthen interpretation of the selected promoter, they do not fully define the relative contribution of each response element under all conditions. More broadly, future optimization of pharmacologically controlled receptor–promoter systems may benefit from more systematic design strategies, including design-of-experiments approaches and data-driven methods such as Bayesian optimization or active learning.

Finally, the in vivo experiments were designed as a proof-of-concept demonstration of pharmacological transgene control using implanted encapsulated cells. While this format offers practical advantages in terms of retrievability and external regulation, its long-term stability, immunological compatibility, and therapeutic performance remain to be established in disease-relevant settings [8,21]. In addition, the in vivo study relied on a short-term reporter-based readout rather than a therapeutic efficacy endpoint. Addressing these issues will be important for future translation of this strategy toward clinically relevant cell-based interventions.

Taken together, the present design provides a pharmacologically controlled addition to the broader inducible gene-switch toolbox. Its main contribution lies not in introducing a fundamentally new signaling principle, but in showing that a human-derived DREADD scaffold can be interfaced with synthetic transcriptional decoders to generate a compact, modular, and pharmacologically addressable gene-control system in mammalian cells and in vivo. This design may be particularly relevant in translational settings where clinically familiar small-molecule control and externally regulated gene expression are both desirable.

## 4. Materials and Methods

### 4.1. Plasmid Construction

All plasmids used in this study are listed in Supplementary Table S1. Designer receptor constructs were generated by fusing the extracellular and transmembrane domains of the human muscarinic M3–based DREADD (hM3Dq) to intracellular signaling domains derived from human β-adrenergic receptors (β1, β2, or β3), as indicated. Synthetic reporter plasmids were constructed by placing the secreted alkaline phosphatase (SEAP) coding sequence under the control of signaling-responsive promoters, including a cyclic adenosine monophosphate (cAMP)-responsive promoter (P_CRE_) [15], an SRE-responsive promoter (P_SRE_), and calcium-responsive promoters (P_NFAT_ and P_CRE-SRE-NFAT_) [19,25]. The annotated sequences of the P_NFAT_-SEAP, P_CRE-SRE-NFAT_-SEAP, P_CRE_-SEAP and P_SRE_-SEAP reporter constructs are provided in the Supplementary Information (Supplementary Sequences S1–S4). The plasmids used in each experiment are summarized in Supplementary Table S3.

Plasmids were assembled using either seamless cloning (homology-directed assembly) or restriction enzyme digestion and ligation according to the manufacturers’ instructions. All constructs were verified by Sanger sequencing prior to use.

### 4.2. Reagents

Cyclosporin A (MedChemExpress, Monmouth Junction, NJ, USA, HY-12026), norepinephrine hydrochloride (MedChemExpress, HY-13715A), histamine (Aladdin, Shanghai, China, H11796), dopamine hydrochloride (Sigma-Aldrich, Saint Louis, MO, USA, H8502), serotonin hydrochloride (TargetMol, Wellesley Hills, MA, USA, T2209), doxycycline (MedChemExpress, HY-N0565-25 mg), acetylcholine chloride (MedChemExpress, HY-B0282-500 mg) and clozapine (MedChemExpress, HY-14539) were dissolved in dimethyl sulfoxide (DMSO; MP Biomedicals, Irvine, CA, USA) to prepare concentrated stock solutions for in vitro experiments and stored at −20 °C. For cell-based assays, stock solutions were diluted into culture medium immediately before use, with the final DMSO concentration not exceeding 0.1% (*v*/*v*).

For in vivo oral administration, clozapine was formulated in a vehicle consisting of 2% (*v*/*v*) DMSO, 40% (*v*/*v*) polyethylene glycol 300 (PEG300, MedChemExpress, HY-Y0873), 5% (*v*/*v*) Tween-80 (MedChemExpress, HY-Y1891), and 53% (*v*/*v*) saline, and administered by oral gavage at the indicated doses. Vehicle-treated control groups received the same formulation without clozapine.

For local topical administration, clozapine was dissolved in ethanol and applied directly to the skin at the implantation site at the indicated concentrations. Ethanol alone was used as the vehicle control for topical treatments.

### 4.3. Cell Culture

HEK293T cells (National Collection of Authenticated Cell Cultures, Chinese Academy of Sciences, Shanghai, China), COS-1 (provided by Procell Life Science & Technology Co., Ltd., Wuhan, China) and human telomerase reverse transcriptase (hTERT)-immortalized cells derived from human mesenchymal stromal cells (hMSC-TERT-derived cells, kindly provided as a gift by Professor Haifeng Ye, East China Normal University) were maintained in Dulbecco’s modified Eagle’s medium (DMEM; HyClone) supplemented with 10% (*v*/*v*) fetal bovine serum (FBS; VisTech™, Sydney, Australia, SE100-B) and 1% (*v*/*v*) penicillin–streptomycin (Biosharp, Beijing, China). CHO-K1 cells (National Collection of Authenticated Cell Cultures, Chinese Academy of Sciences, Shanghai, China) were maintained in RPMI-1640 medium supplemented with 10% (*v*/*v*) FBS and 1% (*v*/*v*) penicillin-streptomycin. Cells were cultured at 37 °C in a humidified incubator with 5% CO_2_ and routinely tested negative for mycoplasma contamination. Where indicated, HEK293T cells were maintained with FBS certified for tetracycline-regulated systems (Vivacell, Shanghai, China, C2720-0050) in place of standard FBS.

### 4.4. Transient Transfection

HEK293T, COS-1, and CHO-K1 cells were transiently transfected using polyethyleneimine (PEI; Polysciences, Warrington, PA, USA, 24765-1). For experiments involving cell encapsulation and in vivo implantation, cells were seeded in 10 cm dishes and cultured to 70–80% confluence prior to transfection. Plasmid DNA was mixed with PEI at a DNA:PEI mass ratio of 1:3 in serum-free medium and incubated for 15 min before addition to cells. After 8–12 h, the transfection medium was replaced with fresh complete medium, and cells were further cultured before encapsulation.

For in vitro screening and functional assays, HEK293T cells were seeded in 48-well or 6-well plates. Transfections were performed using the same PEI-based protocol and DNA:PEI mass ratio, with reagent volumes scaled proportionally to the culture format. After 8–12 h, the transfection medium was replaced with fresh complete medium prior to ligand stimulation.

Equal total plasmid DNA was used across comparative conditions, with the corresponding receptor and reporter constructs transfected on matched constitutive/reporter backbones unless otherwise indicated.

### 4.5. SEAP Reporter Assays

#### 4.5.1. Cell Culture Supernatants (Colorimetric)

SEAP activity in cell culture supernatants was measured using a colorimetric assay. Supernatants were heat-inactivated at 65 °C for 30 min. An aliquot of each sample was mixed with assay buffer (2 M diethanolamine, 1 mM MgCl_2_, 2 mM L-homoarginine, pH 9.8) and p-nitrophenyl phosphate substrate. Absorbance at 405 nm was recorded at 37 °C using a microplate reader (Varioskan LUX, Thermo Fisher Scientific, Waltham, MA, USA).

#### 4.5.2. Serum Samples (Chemiluminescence)

Serum SEAP levels were quantified using a SEAP Reporter Gene Assay Kit (Abcam, Cambridge, UK, ab133077) according to the manufacturer’s instructions. Serum samples were heat-inactivated at 65 °C for 30 min prior to analysis, and chemiluminescence was measured on a Varioskan LUX microplate reader. SEAP concentrations were determined from a standard curve.

### 4.6. qPCR Analysis of NFAT Family Transcripts

Total RNA was extracted from HEK293T cells and reverse-transcribed into complementary DNA (cDNA) according to the manufacturer’s instructions. qPCR was performed to detect *NFATC1*, *NFATC2*, *NFATC3*, and *NFATC4*, using *GAPDH* as the internal control. Primer sequences are listed in Supplementary Table S2. Three biological replicates were analyzed. Data are presented as ΔCt values. No reverse transcription (no-RT) controls were included to assess genomic DNA contamination.

### 4.7. Calcineurin Inhibition Assay

HEK293T cells were co-transfected with hM3Dq and P_CRE-SRE-NFAT_-SEAP or transfected with P_SV40_-SEAP as indicated. Cells were pretreated with cyclosporin A (CsA; 2 or 5 μM) for 30 min prior to stimulation with 1 nM clozapine. Reporter activity was measured 24 h later as described above.

### 4.8. Animal Experiments

#### 4.8.1. Ethical Statement

All animal experiments were performed using C57BL/6J mice (male, 6–8 weeks old, 20–25 g). All animal experiments were performed at the Guangzhou experimental animal facility of Lingfu Topu Biotechnology Co., Ltd., Guangzhou, China, a licensed animal research facility, in accordance with institutional guidelines and national regulations for the care and use of laboratory animals. Mice were housed under standard specific-pathogen-free conditions with free access to food and water and were monitored daily. All experimental procedures were reviewed and approved by the Institutional Animal Care and Use Committee (IACUC) of Lingfu Topu Biotechnology Co., Ltd. (approval number: TOP-1PZ-GZ251114).

#### 4.8.2. Encapsulation of Engineered Cells

Engineered HEK293T cells were encapsulated in alginate–poly-L-lysine–alginate (APA) microcapsules using an Encapsulator B-395 Pro (BUCHI, Flawil, Switzerland) equipped with a 200-μm nozzle. Encapsulation parameters were adjusted to generate uniform microbeads suitable for intraperitoneal or subcutaneous implantation.

#### 4.8.3. In Vivo Transgene Induction

For in vivo gene-switch activation, encapsulated HEK293T cells (1 × 10^6^ cells per mouse) were implanted by intraperitoneal or dorsal subcutaneous injection. Following intraperitoneal implantation, clozapine was administered once daily by oral gavage using the PEG300-based vehicle formulation described above. For subcutaneous implantation experiments, clozapine was applied locally by topical administration in ethanol. Control animals received the corresponding vehicle alone. Blood samples were collected 48 h after implantation for SEAP analysis, and serum SEAP levels were quantified as described above.

### 4.9. Statistical Analysis

All quantitative data are presented as mean ± standard deviation (SD) for in vitro experiments and mean ± standard error of the mean (SEM) for in vivo experiments, unless otherwise indicated. Statistical analyses were performed using GraphPad Prism 11 software. The specific statistical tests used, sample sizes, and definitions of error bars are described in the corresponding figure legends. Group sizes were selected based on feasibility and prior experience.

## Data Availability

Data are available from the corresponding author upon reasonable request.

## Supplementary Materials

The following supporting information can be downloaded at: https://www.mdpi.com/article/10.3390/ijms27083381/s1.
