# Supplementary material for: A Clozapine-Responsive GPCR-Based Gene Switch for Pharmacological Control of Gene Expression in Mammalian Cells and In Vivo
Source: Int J Mol Sci. 2026 Apr 9;27(8):3381. doi: 10.3390/ijms27083381 (PMC13116543; doi:10.3390/ijms27083381)
Supplement: Supplementary file 1 [file ijms-27-03381-s001.zip › ijms-4161563-supplementary.pdf]

Supplementary Information

A clozapine-responsive GPCR-based gene switch for pharmacological control of gene expression in mammalian cells and *in vivo*

Guanyang Chen†*, Shiting Li*†*, Peng Bai**

†These authors contributed equally

*Correspondence: baipeng@mail.sysu.edu.cn

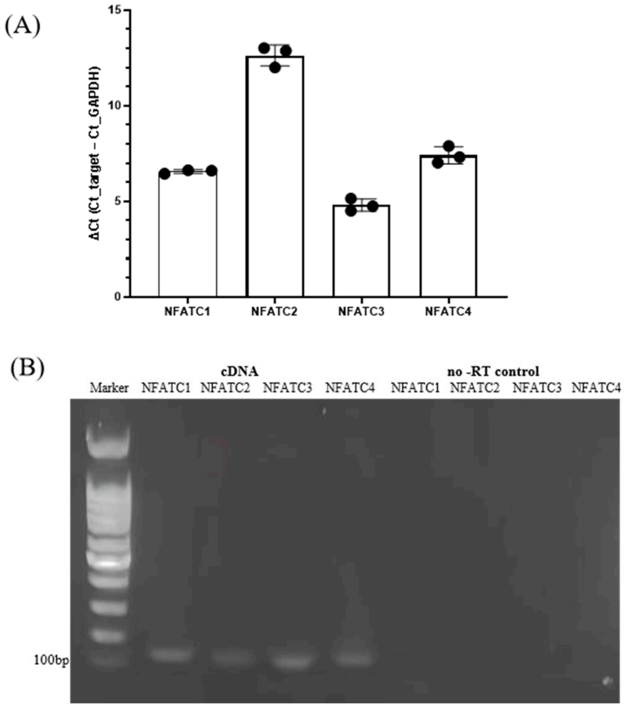

Figure S1. Detection of *NFATC1–C4* transcripts in HEK293T cells.

(A) qPCR analysis of *NFATC1*, *NFATC2*, *NFATC3*, and *NFATC4* expression in HEK293T cells. Data are presented as ΔCt values (Ct_target–Ct_reference). Each dot represents one biological replicate, with technical triplicates averaged before calculation. Bars indicate mean ± SD. Lower ΔCt values indicate higher transcript abundance.

(B) Representative agarose gel image of the corresponding amplicons in cDNA samples and no reverse transcription (no-RT) controls. From left to right: DNA marker, *NFATC1*, *NFATC2*, *NFATC3*, and *NFATC4* amplicons from cDNA samples, followed by the corresponding no-RT controls. No obvious corresponding bands were observed in the no-RT controls.

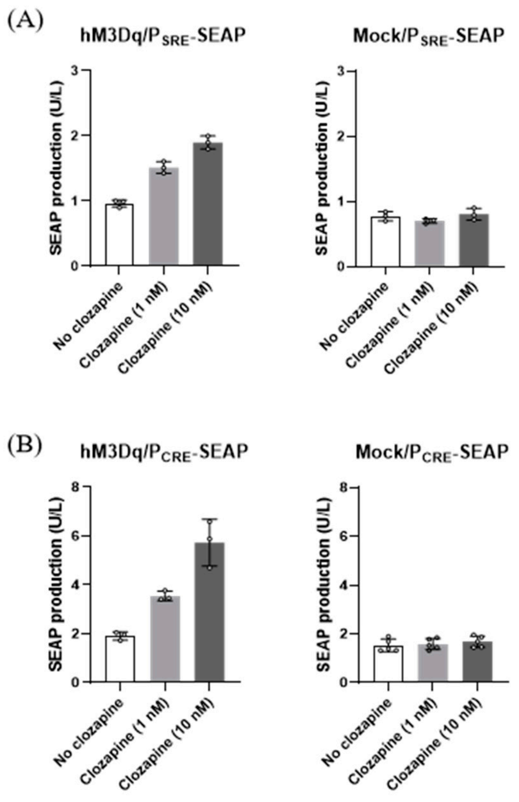

Figure S2. Responses of SRE-element-only and CRE-element-only reporters to clozapine stimulation.

(A) HEK293T cells were co-transfected with P_{SRE}-SEAP and either hM3Dq or the corresponding mock construct, followed by treatment with clozapine at the indicated concentrations. SEAP activity was measured as described above. Data are shown as mean ± SD (n = 3 biologically independent experiments).

(B) HEK293T cells were co-transfected with P_{CRE}-SEAP and either hM3Dq or the corresponding mock construct, followed by treatment with clozapine at the indicated concentrations. The mock group shown in panel B is the same dataset as that presented in Fig. 1a and is repeated here for comparison. Data are shown as mean ± SD (n = 3–5 biologically independent experiments).

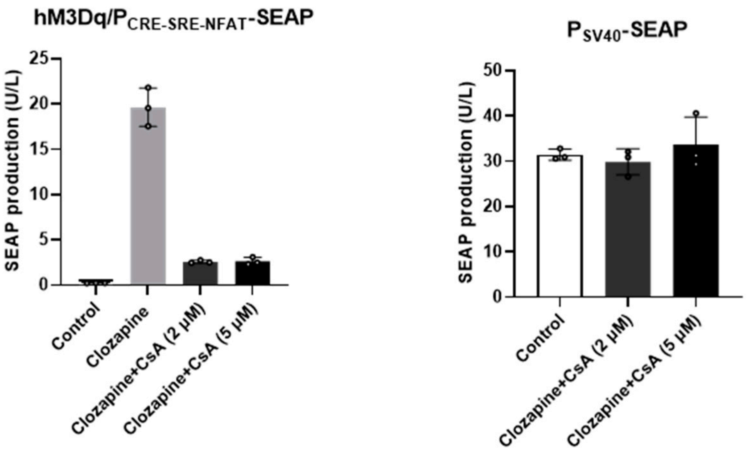

Figure S3. Effect of cyclosporin A (CsA) on clozapine-induced reporter activation.

HEK293T cells co-transfected with hM3Dq and $P_{CRE–SRE–NFAT}$–SEAP were treated with clozapine (1 nM) in the absence or presence of CsA at the indicated concentrations, and SEAP activity in the culture supernatant was measured. HEK293T cells transfected with constitutive P_{SV40}–SEAP were treated under the same conditions, and SEAP activity was measured. Data are shown as mean ± SD (n = 3 biologically independent experiments).

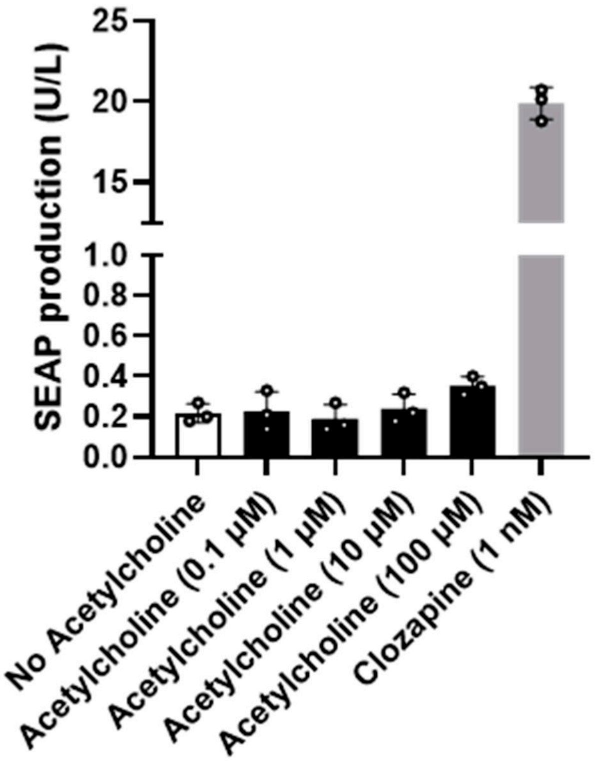

Figure S4. Acetylcholine dose–response analysis of the hM3Dq-based switch.

HEK293T cells co-transfected with hM3Dq and $P_{CRE–SRE–NFAT}$–SEAP were treated with acetylcholine at the indicated concentrations, and SEAP activity in the culture supernatant was measured. Data are shown as mean ± SD (n = 3 biologically independent experiments).

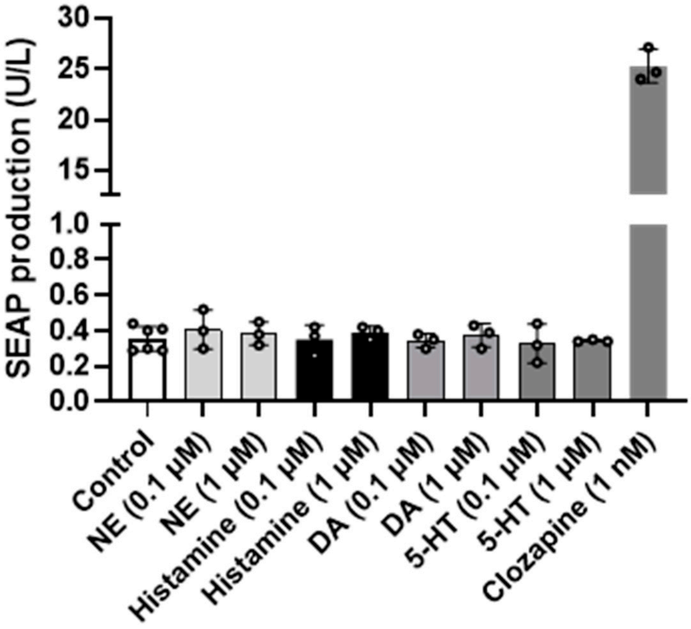

Figure S5. Effects of representative neurotransmitters on reporter activation.

HEK293T cells co-transfected with hM3Dq and $P_{CRE–SRE–NFAT}$–SEAP were treated with norepinephrine (NE), histamine, dopamine (DA), or serotonin (5-HT) at the indicated concentrations, and SEAP activity in the culture supernatant was measured. Data are shown as mean ± SD (n = 3–6 biologically independent experiments).

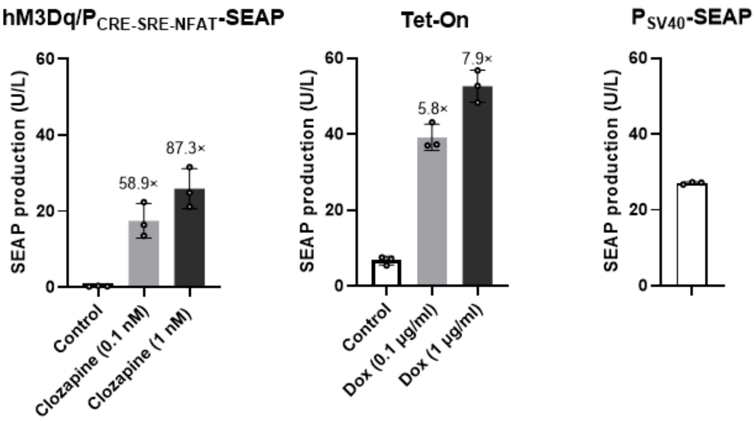

Figure S6. Comparison with Tet-On and constitutive reporter controls

HEK293T cells were transfected with the indicated reporter configurations and cultured in doxycycline-free FBS. Cells were treated with clozapine or doxycycline, depending on the reporter system used. Culture supernatants were collected 24 h later for SEAP measurement. P_{SV40}–SEAP was included as a constitutive reporter control. Fold induction values are indicated in the figure. Data are shown as mean ± SD (n = 3 biologically independent experiments).

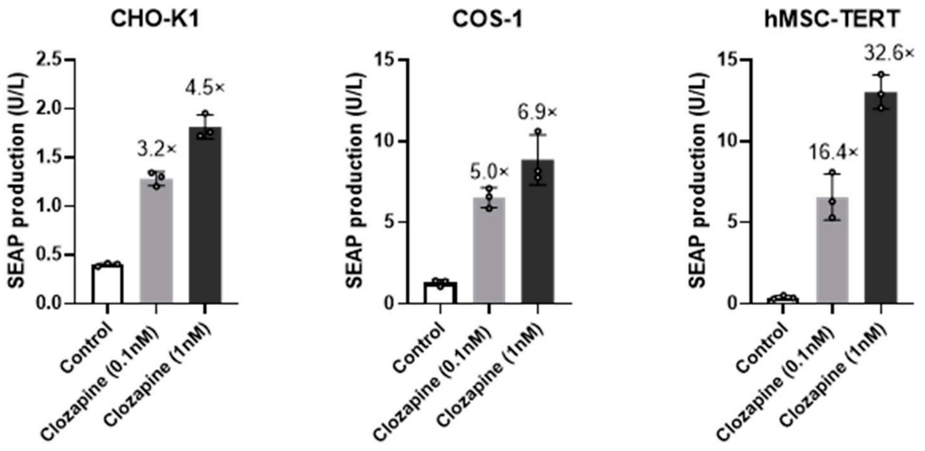

Figure S7. Validation of the hM3Dq-based switch in additional mammalian cell contexts.

The indicated cell lines were transfected with hM3Dq and the $P_{CRE-SRE-NFAT}$–SEAP reporter construct, followed by treatment with clozapine under the indicated conditions. SEAP activity was measured in the culture supernatant 24 h later. Fold induction values are indicated in the figure. Data are shown as mean ± SD (n = 3 biologically independent experiments).

Supplementary Sequences

Sequence S1. P_{NFAT}-SEAP reporter cassette

The sequence contains three tandem NFAT elements, a downstream minimal promoter region, and the *SEAP* coding sequence.

NFAT elements (3 copies)
GGAGGAAAAACTGTTTCATACAGAAGGCGTGGAGGAAAAACTGTTTCATACAGA
AGGCGTGGAGGAAAAACTGTTTCATACAGAAGGCGT

Minimal promoter region
AGATCTAGACTCTAGAGGGTATATAATGGAAGCTCGAATTCCAGCTTGGCATTCC
GGTACTGTTGGTAAAA

SEAP coding sequence (start codon underlined)
agcttcgaatcgcgaattcgcccacc<u>atg</u>ctgctgctgctgctgctgctgggcctgaggctacagctctccctgggcatcatcccagttg
aggaggagaacccggacttctggaaccgcgaggcagccgaggccctgggtgccgccaagaagctgcagcctgcacagacagcc
gccaagaacctcatcatcttcctgggcgatgggatgggggtgtctacggtgacagctgccaggatcctaaaagggcagaagaagga
caaactggggcctgagatacccctggccatggaccgcttcccatatgtggctctgtccaagacatacaatgtagacaaacatgtgccag
acagtggagccacagccacggcctacctgtgcggggtcaagggcaacttccagaccattggcttgagtgcagccgcccgctttaacc
agtgcaacacgacacgcggcaacgaggtcatctccgtgatgaatcgggccaagaaagcagggaagtcagtgggagtggtaaccac
cacacgagtgcagcacgcctcgccagccggcacctacgcccacacggtgaaccgcaactggtactcggacgccgacgtgcctgcc
tcggcccgccaggaggggtgccaggacatcgctacgcagctcatctccaacatggacattgacgtgatcctaggtggaggccgaaa
gtacatgtttcgcatgggaaccccagaccctgagtacccagatgactacagccaaggtgggaccaggctggacgggaagaatctggt
gcaggaatggctggcgaagcgccagggtgcccggtatgtgtggaaccgcactgagctcatgcaggcttccctggacccgtctgtgac
ccatctcatgggtctctttgagcctggagacatgaaatacgagatccaccgagactccacactggacccctccctgatggagatgacag
aggctgccctgcgcctgctgagcaggaacccccgcggcttcttcctcttcgtggagggtggtcgcatcgaccatggtcatcatgaaag
cagggcttaccgggcactgactgagacgatcatgttcgacgacgccattgagagggcgggccagctcaccagcgaggaggacacg
ctgagcctcgtcactgccgaccactcccacgtcttctccttcggaggctacccccctgcgagggagctccatcttcgggctggcccctgg
caaggcccgggacaggaaggcctacacggtcctcctatacggaaacggtccaggctatgtgctcaaggacggcgcccggccggat
gttaccgagagcgagagcgggagccccgagtatcggcagcagtcagcagtgcccctggacgaagagacccacgcaggcgagga
cgtggcggtgttcgcgcgcggcccgcaggcgcacctggttcacggcgtgcaggagcagaccttcatagcgcacgtcatggccttcg
ccgcctgcctggagccctacaccgcctgcgacctggcgccccccgccggcaccaccgacgccgcgcacccgggttactctagagt
cggggcggccggccgcttcgagcagacatga

Sequence S2. $P_{CRE\text{-}SRE\text{-}NFAT}$-SEAP reporter cassette

The sequence contains a cAMP Response Element (CRE)-containing region, three tandem SRE elements, three tandem NFAT elements, a downstream minimal promoter region, and the SEAP coding sequence.

CRE-containing region
CGCACCAGACAGTGACGTCAGCTGCCAGATCCCATGGCCGTCATACTGTGACGTC
TTTCAGACACCCCATTGACGTCAATGGGAGAACCCCGGTACCGGG

SRE elements (3 copies)
AGGATGTCCATATTAGGACATCTAGGATGTCCATATTAGGACATCTAGGATGTCC
ATATTAGGACATCT

NFAT elements (3 copies)
GGAGGAAAAACTGTTTCATACAGAAGGCGTGGAGGAAAAACTGTTTCATACAGA
AGGCGTGGAGGAAAAACTGTTTCATACAGAAGGCGT

Minimal promoter region
AGATCTAGACTCTAGAGGGTATATAATGGAAGCTCGAATTCCAG

SEAP coding sequence (start codon underlined)
aagcttcgaatcgcgaattcgcccaccatgctgctgctgctgctgctgctgggcctgaggctacagctctccctgggcatcatcccagtt
gaggaggagaacccggacttctggaaccgcgaggcagccgaggccctgggtgccgccaagaagctgcagcctgcacagacagc
cgccaagaacctcatcatcttcctgggcgatgggatgggggtgtctacggtgacagctgccaggatcctaaaagggcagaagaagg
acaaactggggcctgagatacccctggccatggaccgcttcccatatgtggctctgtccaagacatacaatgtagacaaacatgtgcca
gacagtggagccacagccacggcctacctgtgcggggtcaagggcaacttccagaccattggcttgagtgcagccgcccgctttaac
cagtgcaacacgacacgcggcaacgaggtcatctccgtgatgaatcgggccaagaaagcagggaagtcagtgggagtggtaacca
ccacacgagtgcagcacgcctcgccagccggcacctacgcccacacggtgaaccgcaactggtactcggacgccgacgtgcctgc
ctcggcccgccaggagggggtgccaggacatcgctacgcagctcatctccaacatggacattgacgtgatcctaggtggaggccgaa
agtacatgtttcgcatgggaaccccagaccctgagtacccagatgactacagccaaggtgggaccaggctggacgggaagaatctg
gtgcaggaatggctggcgaagcgccagggtgcccggtatgtgtggaaccgcactgagctcatgcaggcttccctggacccgtctgtg
acccatctcatgggtctctttgagcctggagacatgaaatacgagatccaccgagactccacactggacccctccctgatggagatgac
agaggctgccctgcgcctgctgagcaggaacccccgcggcttcttcctcttcgtggagggtggtcgcatcgaccatggtcatcatgaa
agcagggcttaccgggcactgactgagacgatcatgttcgacgacgccattgagagggcgggccagctcaccagcgaggaggaca
cgctgagcctcgtcactgccgaccactcccacgtcttctccttcggaggctaccccctgcgagggagctccatcttcgggctggcccct
ggcaaggcccgggacaggaaggcctacacggtcctcctatacggaaacggtccaggctatgtgctcaaggacggcgcccggccg
gatgttaccgagagcgagagcgggagccccgagtatcggcagcagtcagcagtgcccctggacgaagagacccacgcaggcgag

gacgtggcggtgttcgcgcgcggcccgcaggcgcacctggttcacggcgtgcaggagcagaccttcatagcgcacgtcatggcctt
cgccgcctgcctggagccctacaccgcctgcgacctggcgccccccgccggcaccaccgacgccgcgcacccgggttactctaga
gtcggggcggccggccgcttcgagcagacatga

Sequence S3. P_{SRE}-SEAP reporter cassette

The sequence contains three tandem SRE elements, a downstream minimal promoter region, and the SEAP coding sequence.

SRE elements (3 copies)
AGGATGTCCATATTAGGACATCTAGGATGTCCATATTAGGACATCTAGGATGTCC
ATATTAGGACATCT

Minimal promoter region
AGATCTAGACTCTAGAGGGTATATAATGGAAGCTCGAATTCCAG

SEAP coding sequence (start codon underlined)
aagcttcgaatcgcgaattcgcccacc<u>atg</u>ctgctgctgctgctgctgctgggcctgaggctacagctctccctgggcatcatcccagtt
gaggaggagaacccggacttctggaaccgcgaggcagccgaggccctgggtgccgccaagaagctgcagcctgcacagacagc
cgccaagaacctcatcatcttcctgggcgatgggatgggggtgtctacggtgacagctgccaggatcctaaaagggcagaagaagg
acaaactggggcctgagatacccctggccatggaccgcttcccatatgtggctctgtccaagacatacaatgtagacaaacatgtgcca
gacagtggagccacagccacggcctacctgtgcggggtcaagggcaacttccagaccattggcttgagtgcagccgcccgctttaac
cagtgcaacacgacacgcggcaacgaggtcatctccgtgatgaatcgggccaagaaagcagggaagtcagtgggagtggtaacca
ccacacgagtgcagcacgcctcgccagccggcacctacgcccacacggtgaaccgcaactggtactcggacgccgacgtgcctgc
ctcggcccgccaggaggggtgccaggacatcgctacgcagctcatctccaacatggacattgacgtgatcctaggtggaggccgaa
agtacatgtttcgcatgggaaccccagaccctgagtacccagatgactacagccaaggtgggaccaggctggacgggaagaatctg
gtgcaggaatggctggcgaagcgccagggtgcccggtatgtgtggaaccgcactgagctcatgcaggcttccctggacccgtctgtg
acccatctcatgggtctctttgagcctggagacatgaaatacgagatccaccgagactccacactggacccctccctgatggagatgac
agaggctgccctgcgcctgctgagcaggaacccccgcggcttcttcctcttcgtggagggtggtcgcatcgaccatggtcatcatgaa
agcagggcttaccgggcactgactgagacgatcatgttcgacgacgccattgagagggcgggccagctcaccagcgaggaggaca
cgctgagcctcgtcactgccgaccactcccacgtcttctccttcggaggctaccccctgcgagggagctccatcttcgggctggcccct
ggcaaggcccgggacaggaaggcctacacggtcctcctatacggaaacggtccaggctatgtgctcaaggacggcgcccggccg
gatgttaccgagagcgagagcgggagccccgagtatcggcagcagtcagcagtgcccctggacgaagagacccacgcaggcgag
gacgtggcggtgttcgcgcgcggcccgcaggcgcacctggttcacggcgtgcaggagcagaccttcatagcgcacgtcatggcctt
cgccgcctgcctggagccctacaccgcctgcgacctggcgccccccgccggcaccaccgacgccgcgcacccgggttactctaga
gtcggggcggccggccgcttcgagcagacatga

Sequence S4. P_{CRE}-SEAP reporter cassette

The sequence contains a cAMP Response Element (CRE)-containing region, a downstream minimal promoter region, and the *SEAP* coding sequence.

CRE-containing region
CGCACCAGACAGTGACGTCAGCTGCCAGATCCCATGGCCGTCATACTGTGACGTC
TTTCAGACACCCCATTGACGTCAATGGGAGAACCCCGGTACCGGG

Minimal promoter region
GCCGCCCCGACTGCATCTGCGTGTTCGAATTCGCCAATGACAAGACGCTGGGCGG
GGTTTGTGTCATCATAGAACTAAAGACATGCAAATATATTTCTTCCGGGGACACC
GCCAGCAAACGCGAGCAACGGGCCACGGGGATGAAGC

SEAP coding sequence (start codon underlined)
aagcttcgaatcgcgaattcgcccaccatgctgctgctgctgctgctgctgggcctgaggctacagctctccctgggcatcatcccagtt
gaggaggagaacccggacttctggaaccgcgaggcagccgaggccctgggtgccgccaagaagctgcagcctgcacagacagc
cgccaagaacctcatcatcttcctgggcgatgggatgggggtgtctacggtgacagctgccaggatcctaaaagggcagaagaagg
acaaactggggcctgagataccccctggccatggaccgcttcccatatgtggctctgtccaagacatacaatgtagacaaacatgtgcca
gacagtggagccacagccacggcctacctgtgcggggtcaagggcaacttccagaccattggcttgagtgcagccgcccgctttaac
cagtgcaacacgacacgcggcaacgaggtcatctccgtgatgaatcgggccaagaaagcagggaagtcagtgggagtggtaacca
ccacacgagtgcagcacgcctcgccagccggcacctacgcccacacggtgaaccgcaactggtactcggacgccgacgtgcctgc
ctcggcccgccaggaggggtgccaggacatcgctacgcagctcatctccaacatggacattgacgtgatcctaggtggaggccgaa
agtacatgtttcgcatgggaaccccagaccctgagtacccagatgactacagccaaggtgggaccaggctggacgggaagaatctg
gtgcaggaatggctggcgaagcgccagggtgcccggtatgtgtggaaccgcactgagctcatgcaggcttccctggacccgtctgtg
acccatctcatgggtctctttgagcctggagacatgaaatacgagatccaccgagactccacactggacccctccctgatggagatgac
agaggctgccctgcgcctgctgagcaggaacccccgcggcttcttcctcttcgtggagggtggtcgcatcgaccatggtcatcatgaa
agcagggcttaccgggcactgactgagacgatcatgttcgacgacgccattgagagggcgggccagctcaccagcgaggaggaca
cgctgagcctcgtcactgccgaccactcccacgtcttctccttcggaggctaccccctgcgagggagctccatcttcgggctggcccct
ggcaaggcccgggacaggaaggcctacacggtcctcctatacggaaacggtccaggctatgtgctcaaggacggcgcccggccg
gatgttaccgagagcgagagcgggagccccgagtatcggcagcagtcagcagtgcccctggacgaagagacccacgcaggcgag
gacgtggcggtgttcgcgcgcggcccgcaggcgcacctggttcacggcgtgcaggagcagaccttcatagcgcacgtcatggcctt
cgccgcctgcctggagccctacaccgcctgcgacctggcgccccccgccggcaccaccgacgccgcgcacccgggttactctaga
gtcggggcggccggccgcttcgagcagacatga

Supplementary Table S1. Plasmids used and designed in this study

Plasmid	Description	Reference or source
pST51	Constitutive rM3Ds expression vector (SV40p-rM3Ds-pA)	This study
pST6	Constitutive EGFP expression vector (SV40p-EGFP-pA)	This study
pGY8	Constitutive hM3Dq expression vector (SV40p-hM3Dq-pA)	This study
pGY29	Constitutive hM3Dq-β2 chimeric receptor expression vector (SV40p-hM3Dq-β2-pA); based on a previously reported amino acid-level design [12]	This study
pGY46	Constitutive hM3Dq-β3 chimeric receptor expression vector (SV40p-hM3Dq-β3-pA); based on the amino acid region partitioning reported in [12]	This study
phM3Dq-β1	Constitutive hM3Dq-β1 chimeric receptor expression vector (SV40p-hM3Dq-β1-pA); based on a previously reported amino acid-level design [13]	Commercially synthesized (this study)
pCRE-SEAP	P_{CRE}-driven SEAP expression vector (P_{CRE}-SEAP-pA)	Previously reported [15,18]
pNFAT-SEAP	P_{NFAT}-driven SEAP expression vector (P_{NFAT}-SEAP-pA)	Previously reported [19,25]
pCRE-SRE-NFAT-SEAP	$P_{CRE\text{-}SRE\text{-}NFAT}$-driven SEAP expression vector ($P_{CRE\text{-}SRE\text{-}NFAT}$-SEAP-pA)	Previously reported [19,20]
pSRE-SEAP	P_{SRE}-driven SEAP expression vector (P_{SRE}-SEAP-pA)	This study
pSV40-rtTA3	Constitutive rtTA3 expression vector (SV40p-rtTA3-pA)	This study
P_{TRE3GV}-SEAP	P_{TRE3GV}-driven SEAP expression vector (P_{TRE3GV}-SEAP-pA)	Unpublished
pSEAP2-control	Constitutive SEAP expression vector driven by the simian virus 40 promoter (P_{SV40}-SEAP-pA)	Clontech

Abbreviations: **CRE**, cAMP response element; **SRE**, serum response element; **NFAT**, nuclear factor of activated T cells; **EGFP**, enhanced green fluorescent protein; **pA**, polyadenylation signal; **SEAP**, secreted alkaline phosphatase; **SV40p**, simian virus 40 promoter; **P_{TRE3GV}**, doxycycline-responsive promoter; **rtTA3**, reverse tetracycline-controlled transactivator;

hM3Dq, human M3 muscarinic receptor-based Gq-coupled DREADD; **rM3Ds**, rat M3 muscarinic receptor-based Gs-coupled DREADD; **β1/β2/β3**, β1-/β2-/β3-adrenergic receptor-derived sequences

Supplementary Table S2. Sequences of qPCR primers used in this study

NFATC1-F	CACCAAAGTCCTGGAGATCCCA
NFATC1-R	TTCTTCCTCCCGATGTCCGTCT
NFATC2-F	GATAGTGGGCAACACCAAAGTCC
NFATC2-R	TCTCGCCTTTCCGCAGCTCAAT
NFATC3-F	AGACAGTCGCTACTGCAAGCCA
NFATC3-R	GCGGAGTTTCAAAATACCTGCAC
NFATC4-F	GCACCGTATCACAGGCAAGATG
NFATC4-R	TCAGGATTCCCGCGCAGTCAAT
GAPDH-F	GGACTCATGACCACAGTCCA
GAPDH-R	AGGCAGGGATGATGTTCTGG

Supplementary Table S3. Plasmids used in each figure

**Figure 1**	
a	pST51, pGY29, pGY46, phM3Dq-β1, pST6, pCRE-SEAP
b	pGY8, pST6, pNFAT-SEAP, pCRE-SRE-NFAT-SEAP

**Figure 2**	
	pGY8, pCRE-SRE-NFAT-SEAP, pST6

**Figure 3**	
a-b	pGY8, pCRE-SRE-NFAT-SEAP

**Figure 4**	
a-b	pGY8, pCRE-SRE-NFAT-SEAP

**Figure S2**

	pGY8, pCRE-SEAP, pSRE-SEAP, pST6

**Figure S3**	
	pGY8, pCRE-SRE-NFAT-SEAP, pSEAP2-control

**Figure S4**	
	pGY8, pCRE-SRE-NFAT-SEAP

**Figure S5**	
	pGY8, pCRE-SRE-NFAT-SEAP

**Figure S6**	
	pGY8, pCRE-SRE-NFAT-SEAP, pSV40-rtTA3, P_{TRE3GV}-SEAP, pSEAP2-control

**Figure S7**	
	pGY8, pCRE-SRE-NFAT-SEAP
